# Texture-Modified Food for Dysphagic Patients: A Comprehensive Review

**DOI:** 10.3390/ijerph18105125

**Published:** 2021-05-12

**Authors:** Dele Raheem, Conrado Carrascosa, Fernando Ramos, Ariana Saraiva, António Raposo

**Affiliations:** 1 Northern Institute for Environmental and Minority Law (NIEM), Arctic Centre, University of Lapland, 96101 Rovaniemi, Finland; braheem@ulapland.fi; 2 Department of Animal Pathology and Production, Bromatology and Food Technology, Faculty of Veterinary, Universidad de Las Palmas de Gran Canaria, Trasmontaña s/n, 35413 Arucas, Spain; ariana_23@outlook.pt; 3 Pharmacy Faculty, University of Coimbra, Azinhaga de Santa Comba, 3000-548 Coimbra, Portugal; framos@ff.uc.pt; 4 REQUIMTE/LAQV, R. D. Manuel II, Apartado 55142, 4051-401 Porto, Portugal; 5 CBIOS (Research Center for Biosciences and Health Technologies), Universidade Lusófona de Humanidades e Tecnologias, Campo Grande 376, 1749-024 Lisboa, Portugal

**Keywords:** dysphagia, the elderly, food industry, food products, nutrition, processing, rheology, texture

## Abstract

Food texture is a major food quality parameter. The physicochemical properties of food changes when processed in households or industries, resulting in modified textures. A better understanding of these properties is important for the sensory and textural characteristics of foods that target consumers of all ages, from children to the elderly, especially when food product development is considered for dysphagia. Texture modifications in foods suitable for dysphagic patients will grow as the numbers of elderly citizens increase. Dysphagia management should ensure that texture-modified (TM) food is nutritious and easy to swallow. This review addresses how texture and rheology can be assessed in the food industry by placing particular emphasis on dysphagia. It also discusses how the structure of TM food depends not only on food ingredients, such as hydrocolloids, emulsifiers, and thickening and gelling agents, but also on the applied processing methods, including microencapsulation, microgels as delivery systems, and 3D printing. In addition, we address how to modify texture for individuals with dysphagia in all age groups, and highlight different strategies to develop appropriate food products for dysphagic patients.

## 1. Introduction

Food colloids are multi-component, multi-phase systems, involving a complex mixture of water, proteins, polysaccharides, lipids, and many minor constituents that contribute to food textures [1]. While eating and swallowing food, sensory tasks require the tongue’s motor behavior to explore, squeeze, or move a bolus to ascertain its flow properties [2]. However, eating and swallowing food can pose problems that result in dysphagia; those with this condition are dysphagic patients.

Dysphagia refers to difficulty in swallowing, or sometimes the impossibility of swallow liquid or semisolid/solid food [3]. This condition affects almost 580 million people worldwide, especially infants and the elderly, and it leads to nutritional deficiencies [4,5]. As populations in many developed countries age, the number of dysphagic patients is likely to rise. Approximately 2 billion people will be aged 60 and over by 2050, in many countries, (e.g., Japan, Germany and Korea); around 15% of their populations will be over 80 years old [6]. The older population is the global population’s fastest growing segment. Average life expectancy at birth is expected to rise from the present 70 years to 77 years by 2045, with more than 400 million individuals older than 80 years by 2050 [7]. Hence, urgent attention must be paid to the food and nutrition requirements of the elderly, particularly those who are very old and frail. This creates an excellent opportunity for food scientists to respond by formulating food products that meet this demand [8]. Apart from the elderly, and infants whose muscle mass and strength—related to swallowing foods—are weak, those with other medical conditions, such as trauma, cancer, surgery, cerebral palsy, stroke, and other neurological conditions in any age group, may also suffer dysphagia.

Modifying food texture and liquid thickness, without compromising nutritional quality, will play a key role in dysphagia management to ensure that patients can meet their nutritional requirements [9]. For example, studies on bolus rheology by Ishihara et al. [10] suggest that bolus viscoelasticity balance is important to ease swallowing. Other researchers recommended that food texture for dysphagia diets be soft, smooth, moist, elastic, and easy to swallow [11,12]. Handling viscous food components will involve more studies on their rheological parameters. However, a general understanding of the parameters defining texture-modified (TM) food for dysphagia patients worldwide is generally lacking. One extremely important matter for dysphagia management and treatment is to implement the same terminology for it to be universally accepted. A classification system for food viscosity and texture based on sound empirical evidence to help with dysphagia management is necessary. There is a gap in communicating and collaborating among experts in food services and clinical staff. To bridge this gap, in 2012, the International Dysphagia Diet Standardization Initiative (IDDSI) was founded to provide a globally standardized terminology and definitions for TM food and liquids that are applicable to dysphagia individuals of all ages, in all care settings, and for all cultures [13]. We need to conduct more studies to maintain a valid and quantitatively defined scale for different food/fluid textures that can be tested under clinical conditions. Likewise, developing standard recipes for TM food and fluid is also important. For example, in order to provide foods with suitable textures to dysphagic patients, healthcare personnel will have to communicate what this texture is to food producers.

Texture is a sensory multiparameter attribute. It includes all the attributes of the rheological and structural properties of a food product, perceptible by mechanical, visual, auditory and tactile preceptors [14,15]. The roots of the multiparameter attributes of texture lies in its molecular, microscopic, or macroscopic structure. Moreover, certain texture aspects can be seen by the naked eye (e.g., coarse or fine cake texture) or heard by ears (e.g., sounds made when biting on a crunchy celery stalk or a crisp piece of toast) [16]. Dysphagic patients need nutritious foods; such foods need to be of the right texture to improve their consumption and deliver the required nutrients. The need for better intervention strategies is addressed in previous works that target elderly hospitalized patients; this is important because it has the potential to improve patient treatments and outcomes [17]. There are concerns that some TM strategies, such as the IDDSI, do not address the nutritional aspects of foods [9].

Food industries are concerned about variations in taste that come about with changes in viscosity and flow behavior. For instance, evidence suggests that increasing solution viscosity in regular syrup substantially lowers taste intensity, while an increased non-Newtonian flow property observed in light syrup diminishes taste intensity [18]. A better understanding of rheological properties would allow the systemic development of food products to be designed for desired texture and taste interactions. Texture, for many food materials, is a key quality factor. Knowledge gained from the rheological and mechanical properties of various food systems will be relevant for designing flow processes to ensure quality, and to predict storage and stability measurements. Rheological behavior is directly associated with sensory qualities, which significantly influence taste, mouth feel, and stable shelf life. Hence, there is a need for caterers and food scientists to formulate suitable food products for aging populations, which requires a classification system based on rheological properties, consistency, and texture for dysphagia management. These products can be developed for dysphagic patients by blending food ingredients according to personalized recipes for TM food and fluids [19]. To supply dysphagic patients with appropriately textured food, healthcare personnel have to communicate this texture to food service providers by utilizing the same terminology in dysphagia management and treatment [20].

In order to overcome this dilemma, a guide for TM food was developed, in collaboration with dieticians, speech and language pathologists, and a food company specialized in TM diets (Table 1). The purpose of this guide was to develop different food texture definitions based on several Swedish documents. This guide was influenced by the guidelines developed by the British Dietetic Association in collaboration with the Royal College of Speech and Language Therapists [21,22]. It was a pioneering work in the early years (2000–2002), which objectively defined and quantified categories of texture-modified food by conducting rheological measurements and sensory analyses [23]. However, there are limitations in the work, as analyses were conducted on 15 representative TM sample food items. Moreover, individual medical research will be needed to provide diet recommendations to dysphagic patients.

Today, the literature on the impacts of TM food, developed by food scientists, on food swallowing, remains scarce. Food processing industries are adopting various treatments—including thermal and non-thermal treatments—to modify texture. Future trends will likely include a combination of three-dimensional (3D) printing and drying to design foods, and to enhance textural and sensory characteristics for dysphagic patients [24]. A good starting point to develop these new food products is to gain a better understanding on sensory and rheological characteristics (see Table 1), which will be useful for modifying food texture. The objectives of this review article are to raise awareness about the importance of texture modification in the foods provided to dysphagic patients, describe methods to assess viscosity and texture properties in TM food for dysphagia, and compile those aspects that are related to the nutritional quality of foods for dysphagia. Section 2 describes various textural properties by highlighting methods to assess texture in general, particularly referring to dysphagia. Section 3 describes the varying effects of ingredients and processing methods on food texture. Section 4 discusses texture modification for dysphagic patients. Section 5 offers some food examples developed for dysphagic patients. Finally, Section 6 concludes this review.

## 2. Methods to Assess Texture in the Food Industry

The International Organization of Standardization (ISO) recognizes texture as both a sensory quality attribute and a multiparameter attribute. The commonly accepted ISO defines texture as all rheological and structural (geometric and surface) attributes of a food product perceptible by means of mechanical, tactile and, wherever appropriate, visual and auditory preceptors [14].

Texture and rheology are important parameters that need to be assessed when developing food products. One of the physical properties in food technological and sensory analyses that agrees with the ISO definition related to food texture is given by Szczesniak [25] as: “the sensory and functional manifestation of the structural, mechanical and surface properties of foods detected through the senses of vision, hearing, touch and kinesthetics”. Texture is one of the key food attributes that is used to define product quality and acceptability [26], and even shelf life. This characteristic is present in all food, can affect its handling and processing, and can even be decisive for both shelf life and consumer acceptance. It will depend on the analyzed food type. Thus raw material food, handling and processing conditions, such as storage temperature, can have a significant influence on, for example, meat textural properties [27]. To understand this physical food property, we should understand the role of rheology in food. It was defined by Steffe [28] as “a branch of physics that studies the deformation and flow of matter”. This means that it is the condition under which materials respond to an applied force or deformation, despite the fact that many authors relate rheology to liquid or semiliquid food sensory properties rather than to solids. In cases where swallowing food is difficult, hydrocolloids, which exhibit many functionalities in foods, including thickening, gelling, water-holding, dispersing, stabilizing, film-forming, and foaming agents are useful [29]. They have been used as a texture modifiers in almost all kinds of processed food products [29]. All materials have rheological properties that can be employed to assess raw materials and process characteristics, as well as their behavior and stability, throughout storage time until they are eaten to determine their customer acceptance [30]. This means that rheological analyses are necessary to identify the most suitable foods in accordance with final consumer requirements and to ensure the uniformity of different batches over time [30].

Food rheology has been defined as “the science of the deformation and flow of matter” [31]. Therefore, food texture characterization is no new science. Founded in 1929, the American Society of Rheology has already considered experimental foodstuff rheology [30] and consumers previously employed the food rheology/texture as food quality parameters. Conscientious interest has always been shown in its analysis, which has led it to be characterized in the past with senses by means of sensorial techniques. Nevertheless, this analysis is completely subjective and, thus, it is apt and necessary to perform instrumental analyses, which can relate their results to sensorial tests [32]. 

Matter starts deforming or flowing only when it is acted upon by forces that may be applied deliberately or accidentally; moreover, there is the all-pervading force of gravity that causes “soft” bodies to flow and lose shape. Rheology is, thus, mainly concerned with forces, deformations, and time [31]. Time matters in many ways, but it is often introduced into measuring rates of changes of deformations and forces. The passage of time does not actually bring about changes in materials. Chemical changes in foodstuff often occur with time, but can be studied by rheological methods. Temperature is also important and frequently appears in rheological equations [31].

In 1958, Blair [31] classified the frequently used instrumental techniques to measure texture into three main groups:

- Empirical tests to measure some physical properties under well-defined conditions;
- Imitative tests to simulate the conditions to which a material is subjected in mouths;
- Fundamental tests to measure physical properties, such as viscosity and elasticity.

A widely used imitative test today in the food technology field is the so-called Texture Profile Analysis (TPA). The TPA is not only widespread, but also convenient for rapid food texture evaluations [33], although texture can be measured by expert people with sensorial analyses. This test involves double compression to determine food textural properties. Any food texture identity is rarely a simple matter of understanding a singular characteristic, such as toughness or cohesion. The texture of each food is versatile and related to consumers’ sensory expectations. It is not enough to deliver food with the target hardness and elasticity if consumers do not like it and it does not meet their expectations for that food type [34].

Food oral processing is described as a complex and dynamic pathway that involves mechano/chemoreceptors, mixing with saliva, temperature, friction, etc. When thickening formulas for dysphagia are considered, imitation by means of instrumental techniques is difficult as the physico-chemical features of each specific hydrocolloid or food involved in diet will be differently perceived in the mouth [35]. Viscosity is a fundamental property that is obtained from rheological measurements, and is used as the most important criterion in developing thickeners for dysphagia patients. The American Dietetic Association reached an agreement, which was published in the National Dysphagia Diet T [36], and categorizes foods according to their viscosity (at 50 s^−1^) shear rate range values. The categories are: (1) nectar-like (51–350 cP); (2) honey-like (351–1750 cP); (3) spoon thick (>1750 cP)) to ensure safe swallowing and to facilitate palliative care procedures for different types of patient needs, although the categorization does not consider very relevant sensory aspects. Although viscosity values are obtained at 50 s^−1^, no consensus has been reached by the scientific community about the shear rate value of the swallowing process [36]. A study that considered rheological and tribological responses of biopolymer-based thickening solutions incorporated into different food matrices for dysphagic patients observed that an increase in the biopolymer concentration significantly affected rheological properties as xanthan gum showed the highest viscosity, pseudoplasticity, and viscoelasticity, followed by flaxseed gum [37].

ISO 11036:2020 [38] sensory profiling methods can be used for these attributes. This ISO document specifies a method for developing a texture profile of food products (solids, semisolids, liquids). This method is one approach to the sensory texture profile analysis. Chemical composition determines the basic physical structure of foods which, in turn, influences their texture. An understanding of textural properties will, therefore, require studying the physical structure of foods. Other methods based on physical structure that can offer a description of a product’s textural attributes include light and/or electron microscopy, and an X-ray diffraction analysis that provides information about crystalline structure. Differential scanning calorimetry provides information about melting, solidification, and other phase or state transitions, while a particle size analysis and sedimentation methods offer information on particle size distribution and particle shape [39]. Conventional profiling via QDA^®^, flash profiling and projective mapping performed by panels were used by Albert et al. [40] to describe foods with complex textures. The application of QDA^®^, flash profiling and projective mapping using panels with different degrees of training helps to overcome issues in the sensory description of served hot food with a complex texture [40].

However, a qualitative empirical method on test conditions that can better measure viscosity is lacking, as the literature on dysphagia indicates [41]. Researchers have used a quick empirical test, the line spread test (LST), to compare relative viscosities of several similar products. It measures the consistency of a liquid using the distance that a standard amount of liquid spreads over a horizontal surface when released from a confined chamber [42].

Dr. Szczesniak developed and improved sensory descriptions for the texture of specific food while searching for more universal descriptors to be applied to a broader array of food. One of the goals was to develop a common lexicon and a set of procedures to allow objective and repeatable sensory texture evaluation tests to be run in different laboratories, with several operators, and for many distinct food types [34]. These experiments described and introduced food sensory analyses as five basic independent mechanical parameters: hardness, cohesiveness, adhesiveness, viscosity, elasticity, and into three more dependent parameters (brittleness, chewiness, gumminess) [34]. These mechanical parameters [34] can be read from the curve and compared to the observed sensory characteristics. A high correlation between the measurements taken by this technique and sensory evaluations has been shown.

**Figure 1:**
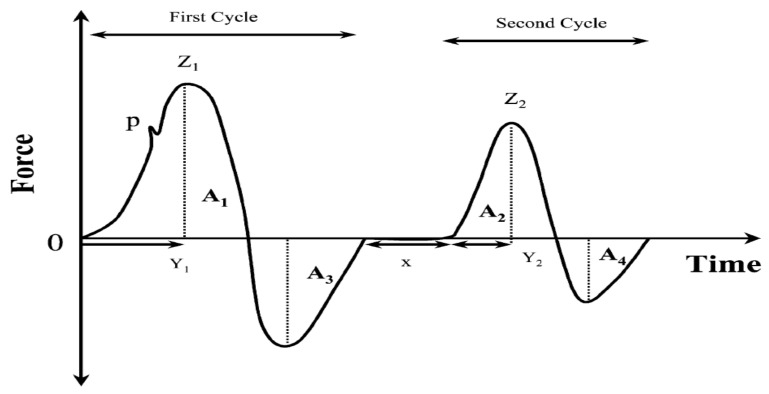
Generalized instrumental texture profile curve modified from Szczesniak [25]. A_1_: positive force area during first compression; A_2_: positive force area during second compression; A_3_: negative force area during first compression; A_4_: negative force area during second compression; Z_1_: height of the maximum force during first compression; Z_2_: height of the maximum force during second compression; Y_1_: time of maxime force during first compression; Y_2_: time of maxime force during second compression; x: time between the first and second compression.

Figure 1 shows a typical TPA graph for food, which is a popular double-compression test run to establish textural food material properties [25] and to quantify mechanical parameters from recorded force-deformation curves. 

Generally, the parameters observed in the texture profile analysis, i.e., hardness, adhesiveness, and cohesiveness, are used to compare the sensory attributes and rheological properties of various foods. They are employed to examine the material properties of commercial oral moisturizers and denture adhesives, which are relevant to dysphagia [43].

In the curves generated in the two TPA cycles, when foods are chewed over time, as shown in Figure 1**,** hardness (N/m^2^): the peak force in the first compression cycle (Z_1_); adhesiveness: negative force area A_3_ for the first bite; cohesiveness (J/m^3^): the ratio between the positive force area during the second compression and that during the first compression (A_2_/A_1_); springiness: Y_2_/Y_1._

It is important to emphasize that TPA has not been broadly used in texture measurements for dysphagia as it does not assess some of the core attributes that are relevant to their foods, which are important; they include slipperiness, humidity, and mouth coating. However, other new tests were developed to help complete the knowledge about the physicochemical properties of food in different analytical fields, such as microscopic, submicroscopic, and molecular [44]. This technical progress was assisted by computer science. Indeed, without computer aid, modern spectroscopy, calorimetry, microscopy, and rheological equipment would not have been able to help texture analyses [44].

Nowadays, new texture analyses include a range of food texture-related parameters: firmness, hardness, consistency, fibrosity, tenderness, elasticity, resistance, gel strength, stickiness, adhesiveness, spreadability, bloom force, extensibility, cohesiveness, chewiness extrudability, texture profile analyses, rubberiness, and resilience. Touch characteristics can be classified as mechanical, which measure chewing effort, geometric, related to shape, and others, such as moisture and fat content. Therefore, most of these characteristics are perceived in the mouth if we bear in mind that texture includes all the steps from the first bite to swallowing [45]. Food mastication covers different processes, including deformation and flow (rheology), size reduction (comminution), and mixing and hydration with saliva. Other physical behaviors that can also be relevant for texture are changes in temperature and surface roughness (rugosity). Food researchers should run rheological tests to describe only a portion of the physical properties sensed in our mouth while chewing [46].

An assessment of rheological properties, particularly in relation to the dysphagia field, includes tests on the flowability or consistency of food. For these tests, a Bostwick Consistometer can be applied to assess the slump of sauces and condiments using a volume of 75 mL, which is released to flow along a channel. The distance traveled by the liquid over 30 s is used to classify consistency [47]. An adaptation of slumping with a reference to dysphagia drinks is called the line-spread test. The IDDSI flow test can be applied using a standard 10 mL Luer slip tip syringe as the “funnel”. This test classifies consistency based on the volume of the residual liquid in the syringe after a period of a 10 s flow. The resulting levels are then defined as level 0 thin (0–1 mL liquid remaining), level 1 slightly-thick (1–4 mL), level 2 mildly thick (4–8 mL) and level 3, moderately thick (8–10 mL) [48]. The new International Dysphagia Diet Standardization Initiative (IDDSI) classification system considers practical measurements for liquids that could be used in kitchens, bedsides and in laboratories. In addition, devices capable of modeling human swallowing will provide more accurate measurement information on shear rates during swallowing in dysphagic patients. Clinicians can employ either manometry or video-fluoroscopy for this purpose. With a manometry, a probe is inserted into the patient’s pharynx, which obstructs the bolus flow and causes discomfort [49]. During video-fluoroscopic analyses, swallowing of fluids is monitored by X-ray imaging and the entire swallowing process is recorded, which, therefore, enables the examiner to follow the swallowing sequence frame by frame [50].

Studies about food texture rheological properties have been systematically conducted since the early 1950s, while the rheological properties of several food types have been studied, and are summarized in many publications, e.g., for roast turkey breast muscle [51]; Japanese sweets [52], the rheology of food dispersion [53]; and food rheology [54]. Many variables can influence rheological properties, including ripeness, processing methods, temperature, composition, time, instrumental techniques, and analytical assumptions and methods), and modify the results obtained by one test [54]. However, not all tests focus on solving the swallowing problem. Suebsaen et al. [55] prepared banana gels from hydrocolloids for the elderly with dysphagia, modified texture and hardness, to obtain a dessert. This product had different characteristics in instrumental rheology, texture properties, and sensory attributes terms. To improve the swallowing ability of foodstuff, different thickeners are added to normal food and drinks, which may be gum- or starch-based [56].

Food technologists are interested in the mastication process, rheological changes, and other textural properties that occur during this process [46]. For dysphagic patients, sensory tasks that require motor behaviors of the tongue to explore, squeeze, or move a bolus to ascertain its flow properties are challenging tasks related to eating and swallowing foods. In addition to taste receptors in the mouth, trigeminal nerve receptors in the mouth and tongue are capable of detecting both static and dynamic characteristics of items placed in the mouth, such as shape, size, volume, mass, location, temperature, two-point discrimination, and flow or movement [57]. It is, therefore, interesting to understand the sensory function of the tongue for tasks that may be relevant for detecting differences in the flow characteristics of swallowing.

Most of the available information on rheological properties of ready-to-eat dysphagia-oriented products only focuses on viscosity [10]. However, new tests on hardness will be necessary to reveal the effect of elastic modulus on the swallowing ability of solid foods for dysphagia [29]. 

Several aspects could be considered in the foods for dysphagic patients: the positive effect of dysphagia-oriented products on the quality of life of dysphagic patients; improve their nutritional status and prevent more weight loss. Designing standardized diets for each type of dysphagia is proposed as a desirable approach in rheological studies that are related to the management of dysphagia [29].

### 2.1. Gels: Rheological Characterization

Some of the most popular foods, such as gelatin desserts, cooked egg whites, frankfurters, surimi-based seafood analogs, and fruit jellies, can be considered gels. In short, they are solid-in-liquid, and the solid phase immobilizes to the liquid phase [54].

Rheological properties can be measured by (a) puncture test, which is one of the simplest methods to obtain a stress strain curve, and is widely used in both solid and semisolid foods; (b) the torsion test, a method that applies shear stress to samples in a twisting fashion; (c) the folding test, which can be used to measure the binding structure of gels, especially surimi gels, and can be interpreted in cohesiveness terms; (d) the oscillatory test, dynamic rheological testing that evaluates the properties of gel systems, which are suitable for testing the characteristics of gels, gelation, and melting; (e) the stress relaxation test, namely rapid deformation applied to food samples [54]. It can be done while under compression, extension, or shear; (f) yield stress, used for predicting how products respond to processing and/or how they endure performance; (g) rheological characterization of time-dependent fluids, it analyzes the flow behavior (or viscosity) of liquid and semi-liquid food. It is an intrinsic parameter and a measure of fluids’ resistance to flow when shearing stress is applied [54].

### 2.2. Emulsions: Rheology of Food Emulsions

Emulsions are dispersions of one liquid phase in the form of fine droplets in another immiscible liquid phase. The immiscible phases are usually oil and water, so emulsions can be broadly classified as oil in water or water in oil emulsions, depending on the dispersed phase (mild cream, ice cream, butter, margarine, salad dressing, and meat emulsions) [58], although the rheology of food emulsions is mainly dependent on the strength of inter-droplet interactions and dilute emulsions (that is, milk) have a low-viscosity Newtonian behavior. Nevertheless, concentrated food emulsions show gel-like rheological characteristics [59].

### 2.3. Rheological Measurements: Equipment

**Figure 2:**
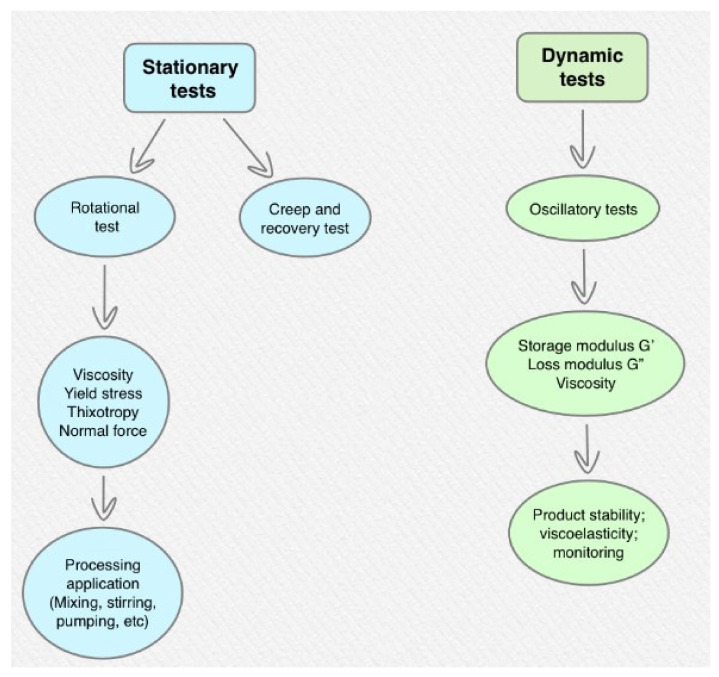
Rheological tests used in food characterization [54].

The rheometer, or viscometer, measures resistance to flow when a known force or stress is produced by a known amount of flow, and is crucial equipment in food rheological studies. Such equipment can be capillary viscometers, falling-ball viscometers, and rotational and oscillatory rheometers, which are used to take rheological measurements [54]. Tests must be carried out under certain conditions for samples, such as steady flow, laminar flow, and uniform temperature [60]. Figure 2 shows the different tests used in rheology.

These rheological measurements use experimentation with and observation of sampled food to compare data, whose main goals are to analyze materials’ mechanical properties and identify molecular interactions and foodstuff composition [61]. Nevertheless, these results should be tested with people. Currently, there are two method groups for rheological studies [29], and both are based on measuring force and deformation according to time:

- Empirical: instrumental-dependent and specified to test a hypothesis;
- Fundamental tests: based on known concepts and equations of physics and fundamental rheology. The European Society for Swallowing Disorders (ESSD) stressed in its published White Paper the importance of rheological parameters, such as shear rate, non-Newtonian fluids properties, yield stress, elasticity, and density [50].

Lack of oral cavity control, poor bolus preparation, or a delayed swallowing response are some reasons for using thicker food and drink for dysphagic patients because thickened foods change the speed at which they are transported through the throat, which is related to delayed swallowing response and, therefore, reduces the aspiration risk [62]. Thickeners, which are typically gum- or starch-based, are added to food to slow down the flow of the bolus. Thickened liquids are highly recommended for dysphagic patients as slowing down the flow rate can provide the time required to close airways [63]. However, excessively thickened food may require much more force on the tongue and pharynx during swallowing [63].

Moreover, we should take into account the texture profile panel, which is a valuable tool for describing and quantifying textural characteristics of food products when the panel is carefully selected, trained, and maintained [64]. Nor should we forget the application from trained panelists or consumer panels in these tests. Thus Saldaña et al. [65] obtained suitable results when sensory hardness correlated positively with instrumental springiness in light mortadella analyses. Other authors, such as Yates [66], performed a descriptive analysis of Gouda cheese texture by a sensory panel and Barden et al. [67] did so on cheddar cheese.

## 3. The Effects of Processing Methods and Ingredients on Food Texture

The structure of modified foods depends very much on the ingredients making it up, and also on the processes involved in their development [68]. Based on these premises, it should be noted that the main building blocks for developing most TM food items are carbohydrates, lipids, and proteins [69]

When heated, globular proteins unfold and denature, which increases liquid viscosity (e.g., in protein drinks). They can self-assemble as nano-sized aggregates and fibrils upon additional heating, and eventually become the network chains of gels [70]. Proteins are appreciated not only for these structural applications, but also for certain essential amino acids (e.g., leucine), whose high hydrolysate content tends to facilitate muscle protein synthesis during aging [71].

Polysaccharides have been used as gelling agents to thicken aqueous food dispersions, and to stabilize emulsions and foams [72]. Nishinari et al. [73] offer an exceptional overview of the rheological properties of polysaccharide solutions and gels associated with tasting and swallowing of TM foods. Dextrins include viscous clear solutions that are often employed as thickening agents and encapsulating matrices for nutrients, colorants, flavors, enzymes, and antioxidants, along with starch and gum [74]. Dietary fiber, such as cellulose derivatives (e.g., microcrystalline cellulose), or resistant starch, which may alleviate constipation, can be added directly to food [75]. Although typically used to thicken liquids, starch has been underexploited as a texture-modifier in the pastes and gels utilized as TM food [76]. As starch granules accumulate large quantities of water during gelatinization, they can be preloaded during this process with water-soluble micronutrients and bioactives [77]. Starch can also be partially gelatinized so that various glycemic responses are elicited [78].

Given their amphiphilic nature, phospholipids and monoglycerides can be used as emulsifiers in interfaces or self-associated with a plurality of nano-sized structures (e.g., vesicles and micelles) as bioactive and nutrient delivery vehicles. Triacylglycerol molecules crystallize from a molten state and cluster to form aggregates and, ultimately, a fat network to occlude parts of liquid fat, which ends in a plastic matrix [79]. Food nano- and micro-emulsion, a topic reviewed recently by McClements [80], can be used to encapsulate and deliver hydrophobic components, such as nutraceuticals, vitamins, and flavors. Oleogels, formed by a liquid lipid process trapped inside a stable gel network, are involved in drug delivery applications as carriers of unsaturated fats and increase food texture [81].

In order to develop TM foods, with a view to retain the overall flavor and appearance of whole pieces while softening their structure, several known technologies achieve this texture-softening effect: freeze-thawing (with/without enzyme infusion), enzyme impregnation, high-pressure processing, pulsed electric fields, and sonication [6]. The regular supervision of process variables maintains the color and flavor of food products, while adjusting their soft texture to various degrees.

Many technologies contribute to small particles and may have applications in TM foods. The food industry has long since been aware of the aggregation and microparticulation of proteins and products employed as thickening agents and fat replacers for beverages and semisolid food [82]. Technologies focus primarily on globular proteins’ capacity to undergo denaturation and aggregation in solution, which results in several morphologies (e.g., spherical particles, fibrils, and flexible strands), whose main dimensions range from approximately 10 nm to a few microns [83].

The aim of another group of techniques is developing fibers and soft particles from biopolymer solutions. Microgels are small soft and stable particles (e.g., sizes from <1–100 mm) and come with a wide variety of shapes, sizes, and textural properties that can be tuned to structures [84]. Microgel formation is often performed by direct gelling, often under shear, in a particle or fiber shape, or by reducing bulk gel size by mechanical means. Microgel suspension is typically free-flowing as opposed to bulk gels with prevailing viscoelastic behavior [85].

Apart from their function as texture modifiers, microgels have been proposed as delivery vehicles for non-polar compounds, such as vitamins, flavors antimicrobials and antioxidants, which can be spread in tiny micelles or more functional liposomes (20 nm and a few hundred mm) in the aqueous phase [86]. Given their soft texture and flowability, micron-sized hydrocolloid gel particles with their high water content (e.g., >95%) are very appealing to be employed as structuring agents to consolidate dispersed phases, and also as soup and sauce thickeners. These hydrocolloid microparticles are generally formed by shear gelling or preformed droplet gelation [87]. A recent study showed that a combination of 0.5% Alcalase, and two-step heating at 37 °C and 90 °C was useful for improving the physico-functional properties of a novel surimi gel for people with dysphagia [88].

Some innovative micro-technology techniques have recently emerged and may lead to revolutionary TM food design and manufacturing applications [89,90,91,92,93,94,95,96,97,98,99]. In channels with cross-sections of a few hundred microns, microfluidic systems handle minute quantities of fluids. Systems have been developed to produce foams and emulsions of identical size and different shapes with a monodispersed discontinuous phase and gel microspheres [92]. 3D printing is a rapid prototyping technique based on digitally-controlled material depositing and layer-by-layer stacking. From “printable” mixtures of carbohydrates it is possible to obtain lipids and proteins, and complex food structures based on liquid deposition or powder binding. According to Kouzani et al. [100], 3D printing reduced design, and fabrication time, improved the consistency and repeatability of 3D printed tuna fish (consisting of tuna, puréed pumpkin, and puréed beetroot), and optimized sensory characteristics of this puréed food for dysphagic patients. Electrospinning employs a high-voltage electrical field to create biopolymer solution electrically-charged jets that become nanofibers upon solvent evaporation. During the encapsulation of bioactives and probiotics, electrospun protein fibers (e.g., <1 µm in diameter) are used as dietary supplements, and also confer food mouthfeel and texture. The nanofibers employed to encapsulate bioactives or as entangled mats to simulate meat are suggested electrospinning technology applications to manufacture TM food [96,97,98]. Electro-spraying is another electrohydrodynamic manufacturing technique whereby near-spherical droplets are produced from a jet flowing through a nozzle submitted to an external electrical field that yields micro- or nanoparticles upon solvent evaporation. Microencapsulation matrices used to protect biologically active compounds is a suggested use for electro-spraying technology in TM food manufacturing [99].

The relevant role thickeners play in TM food while swallowing, slowing down the flow of liquids and stopping them from being aspired via the airway is highlighted [4]. Currently, starch and gums are the most popular commercial choices. Thus increasing the availability of thickeners to be employed in TM food and extending their properties can be challenging [29].

Gel microparticles are excellent alternatives to tailor food rheological properties thanks to their small tunable size, soft texture and free-flowing state [101]. To be able to change their texture perception and flow behavior, they can be blended into thin liquids or incorporated into purées. They evoke a stronger aroma during mouth breakdown if filled with flavors and supplied with a thin delicate texture [102]. Artificial caviars introduced by molecular cuisine proved to be the most innovative use of soft gel particles. Tiny spheres with a soft core and a tough outer layer were formed by dipping droplets in a calcium bath of colored and flavored alginate solutions [103]. Artificial caviars are now often featured in main dishes, desserts, drinks, etc., and are offered in contemporary restaurants [104,105]. Using tiny “gelatinous” beads and other light-molecular cooking creations (i.e., foam or “air”) has been proposed to inspire the elderly to produce attractive TM foods [106].

Recently, the extensive literature on gel microparticles essentially endorses employing them as encapsulating agents and delivery systems rather than applying them to alter texture or to act as major nutritional functions [107,108]. For example, by adding protein microparticles, the texture control of matrices can be achieved [109] and elderly people are likely to try protein-enriched foods if they need a higher protein intake [110]. Conversely, by introducing a dispersed gas phase into bubble form, softness and density can be adjusted [111], which provides the added beneficial effect of a higher perceived intensity of tastants in the gel phase [112]. Insoluble fiber can be filled with gelled microparticles to increase fecal bulk and to prevent constipation, while partially masking the insipid fiber flavor and its rough texture [75,113]. Lastly, emulsion gels are food items in which lipid droplets are enclosed inside a soft biopolymer matrix (e.g., sauces, yogurt, frankfurters, etc.). Gelled emulsion microparticles are small biphasic structures in which a lipid phase offers many opportunities [114]. The incorporation of whey protein isolate (WPI)-based gelled microspheres loaded with lipids into food bars, soups, and other food systems has been suggested by Egan et al. [115]. These microparticles can also be employed as delivery systems for bioactive lipophilic ingredients (fatty acid ω-3, phytosterols, carotenoids, etc.), tastants, and fat-soluble aromas [116]. WPI microgels can lower the plasma insulin peak and postpone the postprandial amino acid profile in relation to protein powder in the interface with drugs [117].

## 4. Modifying Texture for Dysphagic Patients

Food contains several phases and hierarchical structures that vary from nanoscopic to microscopic length scales [118]. The configurations offer some features like texture control and nutritional value, or support for processing and shelf-life stability [101]. Texture control and alteration are common ways to control dysphagia. Modified diets are believed to minimize the risk of choking and the need for chewing or oral food processing [119]. Eating thickened fluids is indicated to help safe swallowing as the act of swallowing is delayed and the transit time of food with an altered consistency in modified foods is typically longer than for non-modified foods. This gives the glottis more time to close and avoids food or fluid aspiration to the lungs of dysphagic patients [120].

Food texture can be modulated and altered to meet consumers’ nutritional demands. Texture modification and thickening of fluids are normal features of dysphagia evaluation and therapy [121]. TM foods can be defined based on many variables, such as viscosity, density, and fluid flow rate. However, using viscosity to describe thickened beverages for dysphagia management has been questioned as no viscosity measurements are available for most clinicians and caregivers [122].

When designing healthy foods for the elderly, significant factors need to be addressed. TM foods prescribed for seniors’ dysphagia management and dietary intake should be soft, moist, smooth, elastic, and simple to swallow [5]. One important key for designing texture and bolus rheology is understanding dynamic food structure changes during oral processing. This rheological state should allow the more cohesively mass flow of bolus throughout the pharyngeal phase to help to improve easy swallowing in dysphagic patients [10].

**Figure 3:**
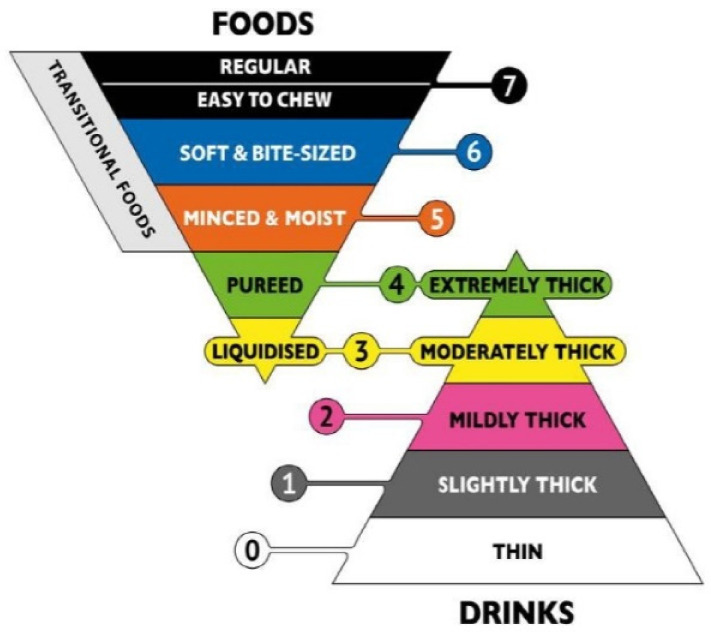
The IDDSI Framework for the TM food and thickened liquids used with dysphagic individuals from all age groups, in all healthcare facilities and of all cultures [122]. Note. 0: thin; 1: slightly thick; 2: slightly thick; 3: liquidized/moderately thick; 4: puréed/extremely thick; 5: minced and moist; 6: soft and bite-sized; 7: easy to chew/regular.

The IDDSI framework provides standardized terms and descriptions to classify TM food and thickened liquids for dysphagia patients [122]. The IDDSI framework consists in a continuum of eight levels (0–7), as shown in Figure 3. In addition, the syringe flow test classifies IDDSI levels from 0 to 3 based on the flow rate, while a fork pressure test is best used to assess the foods of levels 4 to 7 [5].

It is worth noting that foods classified as levels 4 to 7 are texture-modified foods for dysphagic patients [5]. Sungsinchai et al. [5] described the various levels as follows: puréed foods at level 4 do not require chewing, and include products like potato purée, carrot purée, and avocado purée; level 5 (minced and moist) represents soft and moist food with no separate thin liquid; small lumps (of 2–4 mm in size) may be visible in food and minimal chewing is required. Level 5 foodstuff includes items like minced meat and fish, mashed fruit, fully softened cereal, and rice (not sticky or glutinous); level 6 (soft and bite-sized) food that can be mashed and broken down by applying pressure with forks, spoons, or chopsticks that are soft, tender, and moist throughout, but with no separate thin liquid. Chewing is required for this food class, which include cooked tender meat, cooked fish, and steamed or boiled vegetables. Level 7 is regular food with various textures (that can be hard, crunchy and naturally soft).

An example of the TM foods defined in the IDDSI framework is puréed food at the fourth level of the IDDSI framework. Puréed foods are typically ground and/or mixed in a form that involves less chewing and oral manipulation. A cohesive swallowable mass, referred to as ‘bolus,’ is formed that is easy to push with the tongue to the pharynx [123], which can make swallowing simpler and avoid bolus regurgitation, which causes dysphagia aspiration. Other examples of dysphagia-specific standardized scales when considering TM foods for dysphagia include the Penetration-Aspiration Scale [124] SWAL-QOL and SWAL-CARE [125], the Dysphagia Outcome Severity Scale [126], and the Functional Oral Intake Scale [127].

Using thickeners to improve bolus viscosity in post-stroke oral dysphagia has been proposed as a countervailing clinical technique against aspiration. Nonetheless, this strategy has been questioned because the number of experiments is limited and methodologies vary [128]. One experiment has indicated improved safe swallowing when patients received altered starch and xanthan gum thickeners with ‘spoon-thick’ viscosity. The therapeutic effect of these thickeners was due to a counterbalancing process that brought about no major change in swallow reaction timing [129]. Another research study revealed that enhanced bolus viscosity promotes safe swallowing and lowers mid-term pneumonia in patients with oral dysphagia [130]. Some studies have demonstrated that elevated viscosity impairs swallowing effectiveness in oral dysphagia by increasing oropharyngeal residue. Other studies argue that the effect of thickeners on swallow reaction physiology is still not fully understood [129]. Analyzing the effect of augmented bolus viscosity on swallowing safety in patients with dysphagia poses a research challenge. However, novel naturally sourced thickeners from food biopolymers are drawing significant attention and enable on-demand dysphagia management, where fluidal food must be adequately thickened for patients. A recent study investigated the rheological behaviors of a novel thickener with a carboxymethylated curdlan potential for dysphagia, which was a traditional food thickener of konjac glucomannan and its mixtures in both water and model nutrition emulsions. It reported both the efficacy and applicability of these thickened fluids and compared them to those of xanthan gum, taken as the reference. It showed that carboxymethylated curdlan, which is similar to xanthan, displayed a unique viscosity-enhancing ability in both water and emulsions, and proved promising feasible as a novel dysphagia-oriented thickener [131]. Furthermore, the modification of viscosity with thickeners was used as a strategy to circumvent oropharyngeal dysphagia patients’ swallowing problems. Generally, the formulations of commercial food products with thickening properties often contain xanthan and starch. However, flaxseed gum was found to improve rheological behavior in liquid foods and can be considered a potential thickener with additional health benefits [132]. These results offer the opportunity to tailor the rheological characteristics of food systems by adding and combining natural ingredients to improve technological and nutritional properties.

Hydrocolloids are often used by the food industry to enhance consistency and cohesiveness, and to reduce TM foods syneresis. Enhanced food consistency and cohesiveness make it safe to swallow [133]. Although all hydrocolloids can be used as thickeners, they are not all capable of forming a cross-linked gel network to be employed to confer modified food solidity. Food mixture thickening that involves hydrocolloids is mainly the result of polymer chains that entangle while their concentration rises. Entanglements in dilute systems are less common, and polymer chains are free to move and viscosity is minimum. After eating thickened food mixtures, saliva dilutes and breaks, which leads to substantially reduced viscosity. Lower viscosity is a problem, particularly when starch-based thickeners are used because saliva contains α-amylase that breaks down amylopectin and amylose [134]. Non-starch gums can be employed to reduce this, even though they do not completely remove undesirable viscosity declination. When non-starch biopolymer gums are resorted to as thickeners, non-specific entanglement can come into play which, above a given concentration, can increase stickiness, which impairs the ability to swallow. Hydrocolloids in TM food have been reported to have an effect on particle breakdown, microstructure, deformation force during mastication, mouth coating, and bolus lubrication [133]. These properties have an implication for oral processing and sensory food perception. Thickened liquids have also been reported to be considerably less palatable than their non-thick counterparts [135]. It is also necessary to produce new thickening agents that are well-defined in terms of sensory properties, and can be employed to enhance swallowing while preserving palatability. This will include a plan to control dysphagia in order to avoid the detrimental effects of decreased palatability and increasing residual viscosity when complying with therapy. In order to increase palatability, TM foods need to be homogeneous in appearance, and particular attention must be paid to their flavor and odor. Adapting the sensory characteristics of dishes to dysphagia in association with cerebral palsy was possible by the check all-that-apply (CATA) method [136]. CATA is faster, more economical, and does not require trained judges. It is sufficiently robust to obtain the profile of a wide range of food products to be developed.

When modifying texture for dysphagic patients, the influence of two natural different hydrocolloids (apple and citrus pectin) on physical, rheological, and textural parameters, bioactive compounds, and antioxidant activity of courgette (*Cucurbita pepo*) purée was studied. Pectin was added within the 0.1–0.3% range to courgette purée and ohmically heated at 20 V/cm for 3 min. Ohmic heating was utilized to improve and preserve the main properties of purées. Antioxidant activity has also increased with ohmic heating, up to 58% compared to the control sample [137]. The study shows the potential of this treatment for ready-to-eat courgettes as food that can be developed for dysphagic patients.

It should be noted that although several hydrocolloids can be used, they have different physicochemical properties, and even different behavior when preparation variables, such as temperature, shearing, and pressure, are applied.

## 5. Developed Food Products for Dysphagic Patients

People usually eat raw or cooked foods, but swallowing is the key issue. It starts during the mastication process in the mouth, and passes from oropharyngeal safe food transfer to the esophagus to reach the stomach, where the gastro intestinal digestion process starts to allow nutritional food use [138].

Processing food to increase ease of swallowing requires modifications to texture, and also to physicochemical and rheological properties. For instance, plasma processing is effective in improving the cooking properties of brown rice. Swelling of starch granules due to water uptake not only cuts cooking time, but also softens the cooked rice texture and makes it easier to chew. Bran layer fissure significantly improves water absorption and reduces cooking time [5].

Calorie and nutrient requirements diets for dysphagic patients are similar to those presented by persons of the same age and sex, unless co-existent diseases are present [139]. Just like all people, dysphagic patients require suitable food. As previously mentioned in the introduction, this suitability is not only related to food texture, which needs to be appropriate, but should also offer nutritional value and adequate palatability/acceptability and, if at all possible, it must be visually appealing. Combining all of these characteristics is truly challenging [140].

Thus, if we consider not only hospitalized patients, but also the number of older people [141] living in institutionalized settings, and those with dysphagic problems, the major role of food and pharmaceutical industries in developing TM foods is a welcoming development [142,143] The main food texture characteristics that affect dysphagia management can be classified as [138]: adhesiveness (effort made to overcome food adhering to the palate), cohesiveness (if food is deformed or sheared when compressed), firmness (force needed to compress semisolid food), “fracturability” (force required to break solid food), hardness (force required to compress food to attain a certain deformation), springiness (rate or degree that food goes back to its original shape after being compressed), viscosity (rate of flow per unit of force) and yield stress (minimum shear stress applied before flow begins) [134]. The recommended food texture of dysphagic diets must be, at least, smooth, moist, soft and elastic if we contemplate that these attributes should combine TM food rheological properties and patients’ difficulty swallowing [29,119].

The IDDSI framework shown in Figure 3 is a useful guide when a dysphagic patient’s diet is considered. Variations based on individual circumstances may exist. The Functional Diet Scale, in addition to this framework (IDDSI–FDS), permits levels 2–5 as being suitable food and drinks for dysphagic patients. In a Canadian study with adults living in long-term care institutions, IDDSI Functional Diet Scale scores were derived based on diet orders and were compared between residents with and without dysphagia. The IDDSI–FDS for residents with no dysphagia risk ranged from 4 to 8, which reflects the lack of severe diet texture restrictions, while the probability of having an IDDSI–FDS score of <5 was significantly higher in individuals at dysphagia risk [142]. When foods are prepared or formulated for dysphagic patients, it is important for the bolus to be swallowed safely if it is not chewed. Thus particle size and moisture content of food are key criteria, especially for minced and moist foods at level 5. Simple and inexpensive tests at home and in residential care or nursing settings recommended by the IDDSI are: the spoon tilt test to ensure that food is not too dry or sticky; the fork drip test to guarantee that the food is not too runny [9]. In many German nursing homes, minced and moist texture diets are available, which are easy to produce because only a blender and no special knowledge are needed. In these settings, the puréed texture is the most elaborate because they should be lump-free and require special equipment (e.g., a bowl cutter) for several food types (e.g., meat) because of natural fiber content [144].

In one intervention study conducted in a long-term care facility in Canada, the presentation to dysphagic patients of developed foods based on texture and shape resulted in increased body weight, and higher energy and nutrient intake, after 12 weeks in residents receiving reshaped TM diets compared to a control group on unshaped TM diets. Another study in the USA showed a 15% higher food intake after changing to the 3D preparation of puréed foods [145]. This shows that reshaping food components enhances the visual appeal of meals significantly and they are more likely to be eaten. Therefore, it is essential that either liquid or solid food is modified for them to offer appropriate nutritional properties to make swallowing easy for dysphagic patients. Different strategies have been applied to achieve this goal. The main ones can be summarized by following thermal processing and non-thermal technologies, and employing thickeners [139,146].

The simplest form of thermal processing is using hot water, which is known to be effective in transforming hard food into soft food. It is also known that some nutrients are especially heat-sensitive and using thermal processing leads to marked vitamin loss, especially in food rich in these essential nutrients like fruit and vegetables [147]. It is noteworthy that this thermal processing type is often used at home and in industry. To solve this problem, food and pharmaceutical industries usually apply two strategies: first, addition of the micronutrients lost from processing; second, using non-thermal technologies. The most widespread thermal technologies applied to obtain TM food are pulsed electric field, high-pressure processing, high hydrodynamic pressure, ultrasound, and gamma-irradiation [5].

The above-mentioned non-thermal technologies can be applied to meat [148], fish, or its by-products [11], rice [63], starch, and carbohydrate-based products [149], or fruit [150] and vegetables [151]. The use of non-thermal technologies helps to maintain bioactive compounds (especially heat-labile compounds) in food and, thus, promotes health benefits for dysphagic patients. As dysphagic patients benefit from soft food that is safe for swallowing, the characteristics and gel properties of starch play an important role in the desired final product quality.

Irradiation can increase gelatinization temperature, water solubility, water absorption capacity, and oil absorption capacity, but can lower peak, trough, final breakdown, and setback viscosities in starch-based foods. Irradiation has been shown to induce the depolymerization and destruction of the crystalline structure of chickpea flour, which resulted in gamma-irradiated flour being cooked more easily with less retrogradation [5].

The texture of solid food can be generally classified into four grades, as shown in Figure 3. Nevertheless, if regular/unmodified everyday food is not considered, then only three categories are useful for dysphagic patients: (i) “soft”—food is naturally soft (e.g., ripe banana) or cooked or cut to alter food texture; (ii) “minced and moist”—food easily forms into bolus using only the tongue; (iii) “smooth puréed”—food is cohesive enough to maintain its shape on a spoon, similar to the consistency of commercial puddings [152].

No international harmonized terminology is available for thickened liquids, although four or five categories have been defined according to respective viscosity values [4]. However, if the so-called water-like viscosity (<50 cP) is excluded, in a very simplistic form, and according to the “fork test” [63], it is possible to classify thickeners into three texture grades: (i) “nectar”—can be drunk in a cup or with the help of a straw (51-350 cP); (ii) “honey”—can be drunk in a cup, but not with a straw (351–1750 cP); (iii) “pudding”—should be eaten with a spoon (>1750 cP). [4,139].

Commercial TM food was developed to improve nutritional intervention in dysphagic patients. To minimize the risk of aspiration and dehydration, ready-to-serve commercially packaged pre-thickened (CPPT) and instant food thickeners (IFT) are used to modify beverage consistency in dysphagia management [20]. The test of masticating and swallowing solids (TOMASS), an international study that performs quantitative solid bolus ingestion assessments [153], and in vitro testing, such as that performed by Mathieu and co-workers [154] or by Qazi et al. [155], are relevant tools that contribute to assess that these food types do not require further preparation by patients’ families and/or caregivers.

Thus, in commercial terms, food and pharmaceutical companies have made different product types available on the market, which can be summarized as:

(i) Thickeners to be added to liquids and food—the main compounds used to obtain suitable rheological characteristics are gum-based thickeners and starch-based thickeners. The most widespread are carrageenan (E407), modified corn (E1442), xanthan gum (E415), guar gum (E412), and tara gum (E417). Other compounds include calcium citrate (E333) and potassium chloride (E508), used as thickener additives [156,157,158];
(ii) Nutritional supplements with a pudding texture [159];
(iii) Lyophilized or dehydrated powdered products, and pasteurized or sterilized ready to eat or to be reconstituted with both the desired texture, and savory and sweet flavors as purées, or cereals, compotes, and puddings, to eat as breakfasts, snacks, and desserts [139,160]. Examples of commercially developed food products for dysphagia from starch and gum are Nutilis® (Nutricia, Milupa GmbH., Fulda, Germany) and Resource® (Resource, Nestlé Portugal S.A., Linda-a-Velha, Portugal). Both products are presented as white powder that easy to dissolve and can instantly thicken clear liquids. Nutilis® is composed of maltodextrin, modified maize starch (E-1442), tara gum, xanthan gum, and guar gum, while Resource® contains only modified maize starch (E-1442). In both cases, the employed modified starch was hydroxypropyl distarch phosphate [161].

The variety and supply of TM foods targeted at elderly consumers in Asian countries is more promising. The market in Japan is steadily expanding; in South Korea, the market value of the “senior-friendly” food industry in 2010 was around USD 4 million and growing at a rate of 11% per year [6]. The guidelines for TM foods in Japan have been issued by several initiatives, such as food for special dietary uses (FOSDU), the dysphagia diet 2013, and “Smile-Care” foods [6]. Several companies have a special product line that consists mainly in thickened beverages and purées for individuals with swallowing disorders. TM foods offer food companies the opportunity to tailor-make products with soft textures because the products for this market segment have been slow to appear in Europe [162].

Finally, diets should be as varied as possible, and ought to supply sufficient energy and protein. In addition, dishes ought to be pleasantly presented to encourage whetting people’s appetite. Servings should be small and frequent rather than a few copious meals a day. For such purposes, molecular gastronomy [163] and 3D printing technology [100] have been used to produce food from various raw material sources with a variety of textures to enhance diet and to make them more palatable and esthetically appealing. In order to apply 3D printing to food, it is necessary for the food material to possess suitable rheological characteristics to allow its extrusion and for it to be cohesive enough to maintain its shape. However, further research into the application of selected non-thermal technologies as a means to modify food texture for subsequent 3D printing will be worthwhile [5]. In the future, it is envisaged that the food industry will advance toward convergence technology by the utilization of digital solutions, such as machine learning to food systems, as shown in the results of a recent study. This suggests a pioneering framework to identify the rheological levels of foods for the elderly by combining experimental results with machine learning technology in the food application domain [164].

## 6. Conclusions

Food texture modifications are essential to suit the nutritional diets of dysphagic patients. It is necessary to gain a better understanding of the complex factors that influence the colloidal food matrix from a multidisciplinary perspective. Individual and household food service operators, including nursing homes, need to acquire better knowledge about food texture, nutrition, and sensory properties. The elderly and dysphagic patients require a sourcing of special foods that are not only soft and easy and safe to swallow, but are also nutritious and tasty. This is vital for them to achieve their nutritional needs. Current food product development initiatives on the TM foods industrial scale also need to employ novel technologies to ensure dysphagic patients’ access to appropriate TM food products. Additional quality criteria and clinical guidelines that target dysphagic patients, and based on the rheological parameters discussed in this review, need to be introduced by the food industry, and healthcare and catering services. In the very near future, it is hoped that more food processors will engage in the commercialization of cost-effective TM foods to put innovative technologies in this field to the best possible use.

## Figures and Tables

**Table 1:** Descriptions of the consistencies in the texture guide [23].

Category	Description	Example
Regular or cut	Normal texture, possibly cut into smaller pieces.	Whole or cut meat, whole fish, meat or sausage dishes, vegetables, potatoes and gravy. Fresh fruit or canned fruit with whipped cream or ice cream.
Coarse pâtés	Grainy, porous soft texture with coarse grains, such as a juicy and soft meatloaf. Easy to cut with a fork.	Coarse meat pâté or whole steamed fish, coarse vegetable pâté or well-cooked vegetables, whole or pressed potatoes, and gravy. Canned fruit in pieces with whipped cream or ice cream.
Timbales	Smooth, soft, short, and uniform consistency, similar to an omelet. Can be eaten with a fork or spoon.	Meat or fish timbale/soufflé, vegetable timbale/purée, mashed/pressed potatoes, and gravy. Fruit mousse with whipped cream or ice cream.
Jellied products	Soft and slippery food, such as mousse. Can be eaten with a fork or spoon.	Cold jellied meat or fish, vegetable purée or cold jellied vegetables, mashed potatoes, and thick gravy. Jellied fruits with whipped cream or ice cream.
Liquids	Smooth and liquid consistency, such as tomato soup. Fluid runs off the spoon. Cannot be eaten with a fork.	Enriched meat, fish or vegetable soup with whipped cream or crème frâiche. Fruit soup with whipped cream or ice cream.
Thickened liquids	Smooth and viscous, such as sour cream. Fluid drops off the spoon. Cannot be eaten with a fork.	Enriched viscous meat, fish or vegetable soup with whipped cream or crème frâiche. Viscous fruit soup with whipped cream or ice cream.

## Data Availability

Not applicable.
